# Malignant lymphoma of the ureter: A case report and literature review

**DOI:** 10.3892/etm.2014.1658

**Published:** 2014-04-02

**Authors:** BEI-WEN NI, LU ZHONG, TING WANG, FANG-YUAN CHEN

**Affiliations:** Department of Hematology, Renji Hospital, School of Medicine, Shanghai Jiao Tong University, Shanghai 200125, P.R. China

**Keywords:** malignant, lymphoma, ureter

## Abstract

A 38-year-old male was admitted to Renji Hospital (Shanghai, China) with the major complaint of back pain due to left hydronephrosis. Imaging analysis revealed an area of nodular soft-tissue density in the left ureteral wall. The patient’s left kidney was non-functional. Thus, a left nephroureterectomy was performed for the purpose of pathological diagnosis, and histopathological examination revealed follicular lymphoma. The patient received R-CHOP chemotherapy (rituximab, cyclophosphamide, doxorubicin, vincristine and prednisolone) every three weeks. Following six courses of chemotherapy, positron emission tomography-computed tomography revealed that the patient was in complete remission. From this case we showed that in cases where a partial ureteral stenosis with ureteral wall thickening was observed by imaging analysis, further histological examination of tissue samples should be assigned as soon as possible.

## Introduction

Non-Hodgkin’s lymphoma (NHL) has a far greater inclination to disseminate to extranodal sites compared with Hodgkin’s lymphoma (HL). The most common extranodal site is the gastrointestinal tract, whereas NHL of the ureter is rare. Over the past five decades, there have been limited case reports regarding NHL of the ureter. Due to the limited number of cases, treatment of the disease has not been unified. In the current study, a Chinese male with NHL of the ureter was identified. The patient was treated with surgery and chemotherapy. In combination with the relevant literature, the diagnosis and treatment of malignant lymphomas of the ureter are discussed in the present study.

## Case report

### Patient history

In October 2011, a 38-year-old Chinese male complained of back pain and was admitted to Changzheng Hospital (Shanghai, China) with left hydronephrosis, which was confirmed by ultrasound. Ureteroscopy revealed a luminal stenosis of the left ureter, thus, a ureteral double J stent was inserted. A ureteroscopic biopsy was performed and histopathological examination revealed a granuloma. Follow-up examination months later showed no evidence of diminished hydronephrosis. Written informed patient consent was obtained from the patient.

### Patient examination

**Figure 1:**
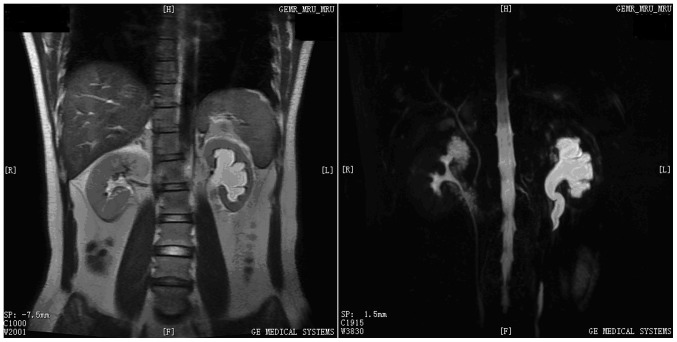
Left hydronephrosis and stenosis of the left ureter as shown by magnetic resonance urography.

**Figure 2:**
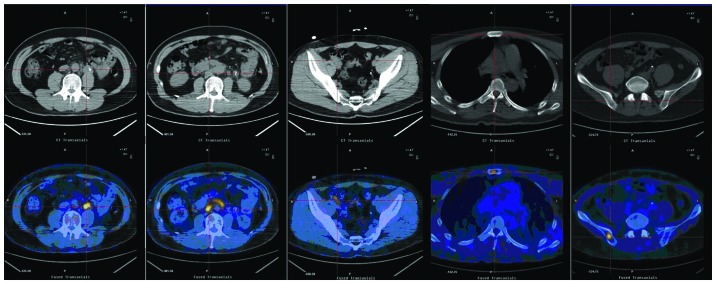
PET-CT scans revealed a nodular soft-tissue density area in the wall of the left-middle ureter at the L3 level, paraaortic and iliac lymphadenopathy and increased FDG uptake in the sternum and right ilium. PET-CT, positron emission tomography-computed tomography; FDG, fludeoxyglucose.

**Figure 3:**
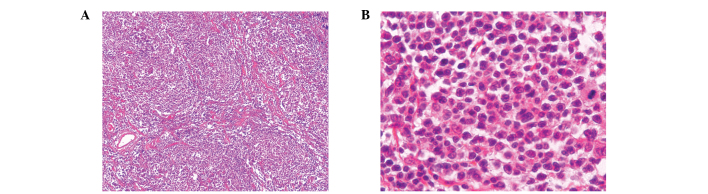
Atypical lymphocytes from follicular nodules, as shown by HE staining at a magnification of (A) ×100 and (B) ×400. HE, hematoxylin and eosin.

**Figure 4:**
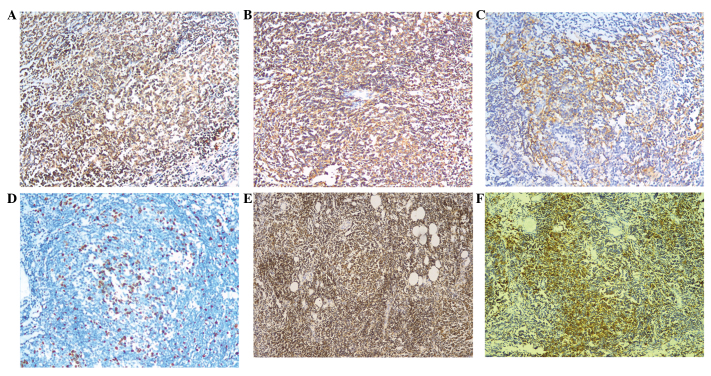
Immumohistochemical staining revealed positive reactions for (A) Bcl-2, (B) CD20, (C) CD21, (D) Ki-67, (E) CD79-alpha and (F) CD23 in the nodular soft-tissue in the wall of the left-middle ureter (magnification, ×100).

In February 2012, the patient was referred to Renji Hospital (Shanghai, China) for further evaluation of the ureteral stenosis with uncertain etiology. Magnetic resonance imaging (MRI) revealed an area of nodular soft-tissue density in the wall of the left-middle ureter (Fig. 1). This section of the ureteral wall exhibited low intensity on T1-weighted images (WIs) and slight hyperintensity on T2-WIs. In addition, MRI scans revealed retroperitoneal adenopathy and no abnormalities in the right ureter. Positron emission tomography-computed tomography (PET-CT) revealed an area of nodular soft-tissue density in the wall of the left middle ureter at the L3 level (Fig. 2). This section had an area of 2.4×2.4 cm, with an average standardized uptake value (SUV) of 6.1. The PET-CT scans also revealed paraaortic and iliac adenopathy with an average SUV of 2.1–5.4. Increased fludeoxyglucose (FDG) metabolism was also observed in the sternum and right ilium (average SUV, 3.4–5.4). Radioisotope renography demonstrated that the glomerular filtration rate (GFR) of the left kidney had significantly decreased to 10.2 ml/min/1.73 m^2^, while the GFR of the right kidney had reduced to 38.3 ml/min/1.73 m^2^. Subsequently, the patient underwent a left nephroureterectomy. Pathological examination revealed that the left-middle ureter exhibited nodular thickening. Additional immunohistochemical analysis of the specimen (Figs. 3 and 4) revealed that the atypical lymphocytes were positive for CD20, CD79α, CD21 and CD23. The Ki-67 index was 10% and Bcl-2 staining was positive in the follicular nodules. Large, atypical cells were not observed and the tumor was diagnosed as a grade 1 follicular NHL. Furthermore, histological examination indicated chronic interstitial nephritis. The bone marrow biopsy appeared normal and the bone marrow cytogenetic study revealed a normal male karyotype.

### Treatment and follow-up

The patient received systemic chemotherapy combining 375 mg/m^2^ rituximab, 750 mg/m^2^ cyclophosphamide, 20 mg/m^2^ liposomal doxorubicin, 1.4 mg/m^2^ vincristine and 80 mg methylprednisolone (R-CHOP), which was administered every three weeks. Following three courses of treatment, PET-CT examination revealed that the paraaortic adenopathy had reduced by 50%, the iliac nodes had disappeared and FDG metabolism in the sternum and right ilium was normal. Following six courses of chemotherapy, the PET-CT scans showed negative for residual tumor. Subsequently, the patient received a further two courses of R-CHOP. Thus far, the follow-up period is seven months and the current evaluation is that the patient is in complete remission.

## Discussion

Clinical manifestation of NHL is diverse and NHL has highly variable outcomes. NHL has a far greater inclination to disseminate to extranodal sites. The extranodal sites normally affected by NHL include the gastrointestinal tract, testes, ovaries, central nervous system, prostate, thyroid, bones and skin. The most common extranodal site is the gastrointestinal tract, whereas NHL of the ureter is rare. An extensive literature search of the PubMed database only identified a few case reports (1–3,5–9) over the past five decades that reported cases of NHL in the ureter. A total of 20 patients with ureteral malignant lymphomas were identified, the majority of which originated from Japan. Detailed information regarding the 20 patients is shown in Table I. The median age of the patients was 56 years, ranging between 12 and 74 years. The majority of the patients exhibited no evident symptoms or only complained of flank pain. Certain patients also presented with hematuria, renal colic or postrenal azotemia. Imaging analysis revealed that the majority of patients had hydronephrosis. The incidence was higher in males, but there was no marked difference with regard to the side or site of the ureteral malignant lymphoma. The most common pathological type was NHL, including diffuse large B-cell lymphoma, follicular B-cell lymphoma, small lymphocytic lymphoma and mucosa-associated lymphoid tissue lymphoma. There were four cases of Hodgkin’s lymphoma among the 20 patients.

There is no particular imaging characteristic and in the majority of cases, diagnosis was established according to the histopathological study of tissue samples obtained from partial ureterectomies or nephroureterectomies. Hashimoto *et al* (6) reported that ureteral mucosal biopsy by ureteroscopy was useful for obtaining enough tissue sample to diagnosis. However, several other reports did not agree, as the vessels and lymphatics of the ureters were longitudinally oriented, thus, determined the direction of further tumor migration (3). Adventitial arterial involvement may allow the tumor to grow away from the wall. In the present case, MRI scans revealed an area of nodular soft-tissue density in the wall of the left-middle ureter. This section of the ureteral wall demonstrated hyperintensity on T2-WIs, which favored a neoplastic disease. PET-CT scans supported this hypothesis. Although an ureteroscopic biopsy was obtained, histological examination revealed a granuloma. Finally, the patient underwent a nephroureterectomy due to the malfunction of the left kidney and thereafter, the diagnosis was established.

Overall, 25–40% of NHL patients present with a primary extranodal lymphoma. However, the definition of primary extranodal lymphoma is controversial, particularly in patients where nodal and extranodal sites are involved. Certain studies have indicated that only patients with localized nidus have primary extranodal lymphoma (10–13). Alternatively, studies that use a more liberal criteria for extranodal lymphoma included patients with a disseminated disease. These two definitions inevitably lead to selection bias. Krol *et al* (14) hypothesized that any lymphoma that is initially clinically dominant at an extranodal site should be considered as a primary extranodal type, even if a disseminated disease was identified. It is difficult to assess whether ureteral lymphomas are primary. Lymphomas usually affect the ureter indirectly with a mass effect caused by adjacent nodal disease. Involvement of the proximal ureter by paraaortic adenopathy and the distal ureter by iliac nodes is typical. However, this situation is not absolute. Although distinguishing whether ureter lymphomas are primary or caused by metastasis is difficult, for the patient in the present study, it was important that the ureter was the initial clinical location of the disease.

In conclusion, in cases where a partial ureteral stenosis with ureteral wall thickening is observed by imaging analysis, further histological examination of tissue samples should be assigned as soon as possible, however, tissue biopsy via ureteroscopy is not recommended.

## Figures and Tables

**Table I:** Summary of reported cases of malignant lymphoma of the ureter.

Age, years	Gender	Symptoms	Side	Site	Pathology	Treatment
52	F	Hematuria	L	U	HL	NU
12	M	Flank pain, hydronephrosis	L	M	HL	NU, R
35	M	Asymptomatic, hydronephrosis	L	L	NHL	NU, Chemo
60	M	Renal colic	R	L	NHL (mixed diffuse and follicular)	Unknown
59	M	Cervical lymphadenopathy	R	U	NHL	Unknown
42	M	Cervical lymphadenopathy	R	U	NHL	Chemo
69	F	Asymptomatic, hydronephrosis	L	M	NHL (follicular)	PU
61	M	Flank pain, hydronephrosis	L	U	NHL (DLBCL)	Chemo
62	M	Postrenal azotemia	Bil	U	NHL (small lymphocytic)	Chemo
52	M	Flank pain, hydronephrosis	L	L	HL	PU, R
41	F	Flank pain, hydronephrosis	R	U	NHL (DLBCL)	Chemo, R
54	M	Asymptomatic, hydronephrosis	R	M	HL	NU, Chemo
72	M	Asymptomatic, hydronephrosis	R	U	NHL (MALT)	PU
58	M	Asymptomatic, hydronephrosis	R	M	NHL (DLBCL)	NU, Chemo
22	F	Flank pain, hydronephrosis	L	L	NHL	PU
71	M	Hematuria	R	M	NHL (follicular)	PU
68	M	Postrenal azotemia	Bil	U	NHL (follicular)	NU, Chemo
28	M	Unknown	Unknown	Unknown	NHL (DLBCL)	Unknown
74	F	Flank pain, urinoma	L	U	NHL (DLBCL)	PU, Chemo
38	M	Backache, hydronephrosis	L	M	NHL (follicular)	NU, Chemo

F, female; M, male; R, right; L, left; Bil, bilateral;U, upper ureter; M, middle ureter; L, lower ureter; NHL, non-Hodgkin’s lymphoma; HL, Hodgkin’s lymphoma; DLBCL, diffuse large B-cell lymphoma; MALT, mucosa-associated lymphoid tissue; NU, nephroureterectomy; PU, partial ureterectomy; Chemo, chemotherapy; R, radiation.

## References

[1] Lebowitz JA, Rofsky NM, Weinreb JC, Friedmann P (1995). Ureteral lymphoma: MRI demonstration. Abdom Imaging.

[2] Chen HH, Panella JS, Rochester D, Ignatoff JM, McVary KT (1988). Non-Hodgkin lymphoma of ureteral wall: CT findings. J Comp Assist Tomogr.

[3] Buck DS, Peterson MS, Borochovitz D, Bloom EJ (1992). Non-Hodgkin lymphoma of the ureter: CT demonstration with pathologic correlation. Urol Radiol.

[4] Curry NS, Chung CJ, Potts W, Bissada N (1993). Isolated lymphoma of genitourinary tract and adrenals. Urology.

[5] Tozzini A, Bulleri A, Orsitto E, Morelli G, Pieri L (1999). Hodgkin’s lymphoma: an isolated case of involvement of the ureter. Eur Radiol.

[6] Hashimoto H, Tsugawa M, Nasu Y, Tsushima T, Kumon H (1999). Primary non-Hodgkin lymphoma of the ureter. BJU Int.

[7] Hara M, Satake M, Ogino H, Itoh M, Miyagawa H, Hashimoto Y, Okabe M, Inagaki H (2002). Primary ureteral mucosa-associated lymphoid tissue (MALT) lymphoma-pathological and radiological findings. Radiat Med.

[8] Kawashima A, Shiotsuka Y, Nin M, Kokado Y (2005). Malignant lymphoma of the ureter: a case report. Hinyokika Kiyo.

[9] Kubota Y, Kawai A, Tsuchiya T, Kozima K, Yokoi S, Deguchi T (2007). Bilateral primary malignant lymphoma of the ureter. Int J Clin Oncol.

[10] Gospodarowicz MK, Sutcliffe SB, Brown TC (1987). Patterns of disease in localized extranodal lymphomas. J Clin Oncol.

[11] Gospodarowicz MK, Sutcliffe SB (1995). The Extranodal Lymphomas. Semin Radiat Oncol.

[12] Paryani S, Hoppe RT, Burke JS (1983). Extralymphatic involvement in diffuse non-Hodgkin’s lymphoma. J Clin Oncol.

[13] Rudders RA, Ross ME, DeLellis RA (1978). Primary extranodal lymphoma: response to treatment and factors influencing prognosis. Cancer.

[14] Krol AD, le Cessie S, Snijder S, Kluin-Nelemans JC, Kluin PM, Noordjik EM (2003). Primary extranodal non-Hodgkin’s lymphoma (NHL): the impact of alternative definitions tested in the Comprehensive Cancer Centre West population-based NHL registry. Ann Oncol.

